# Decline of transmissible gastroenteritis virus and its complex evolutionary relationship with porcine respiratory coronavirus in the United States

**DOI:** 10.1038/s41598-019-40564-z

**Published:** 2019-03-08

**Authors:** Fangzhou Chen, Todd P. Knutson, Stephanie Rossow, Linda J. Saif, Douglas G. Marthaler

**Affiliations:** 1 Department of Veterinary Population Medicine, College of Veterinary Medicine, University of Minnesota, Saint Paul, Minnesota United States of America; 2 State Key Laboratory of Agricultural Microbiology, College of Veterinary Medicine, Huazhong Agricultural University, Wuhan, China; 3 Department of Veterinary Preventive Medicine, The Ohio State University, Food Animal Health Research Program, OARDC, CFAES, Wooster, Ohio United States of America; 4 Veterinary Diagnostic Laboratory, College of Veterinary Medicine, Kansas State University, Manhattan, Kansas United States of America

## Abstract

The epidemiology and genetic diversity of *transmissible gastroenteritis virus* (TGEV) in the United States (US) was investigated by testing clinical cases for TGEV by real time RT-PCR between January 2008 and November 2016. Prevalence of TGEV ranged between 3.8–6.8% and peaked during cold months until March 2013, in which prevalence decreased to < 0.1%. Nineteen complete TGEV genomes and a single strain of *porcine respiratory coronavirus* (PRCV) from the US were generated and compared to historical strains to investigate the evolution of these endemic coronaviruses. Sixteen of our TGEV strains share 8 unique deletions and 119 distinct amino acid changes, which might greatly affect the biological characteristics of the variant TGEV, and resulted in a “variant” genotype of TGEV. The “variant” genotype shared similar unique deletions and amino acid changes with the recent PRCV strain identified in this study, suggesting a recombination event occurred between the ‘‘variant’’ TGEV and PRCV. Moreover, the results indicate the “variant” genotype is the dominant genotype circulating in the US. Therefore, this study provides insight into the occurrence, origin, genetic characteristics, and evolution of TGEV and PRCV circulating in the US.

## Introduction

*Transmissible gastroenteritis virus* (TGEV) belongs to the genus *Alphacoronavirus*, family *Coronaviridae* and contains a single-stranded, positive-sense RNA genome of approximately 28.5 kb in length. The enteropathogenic coronavirus causes watery diarrhea, severe villous atrophy, high mortality in piglets, and severe morbidity in different stages of pig development. In the epidemic form, TGEV causes significant economic losses to the global pork industry^1–4^. In 1946, TGEV was first reported in the United States (US)^5^ and was later identified in Europe (Belgium^6^, England^2^, France^7^, Germany^8^, The Netherlands^6^, and Spain^9^), Asia (China^10^ and Japan^11^), Africa (South Africa^4^ and Uganda^12^), and South America (Brazil^13^). Recent TGEV epidemiological studies are lacking in the US while sporadic outbreaks have been reported from China^14–16^.

Harboring one of the largest RNA genomes, the TGEV genome includes four structural genes (spike [S], envelope [E], membrane [M] and nucleoprotein [N]) and five non-structural open reading frame (ORF): ORF1a and ORF1b, ORF3a, ORF3b, and ORF7. The single segment genome is organized as 5′UTR-ORF1a/1b-S-ORF3a-ORF3b-E-M-N-ORF7-3′UTR^3^. The ORF1a/b can be divided into 15 ORFs, encoding non-structural proteins (NSP)1-10 and NSP12-16. The NSPs contribute to regulation of host translation and localization of the RNA-dependent RNA polymerase (RdRp) during viral replication^3^. In coronavirus, NSP1 is a major factor associated with pathogenicity^17^. The NSP2, 3 and 8 of TGEV can incorporate into the viral articles^18^ while the NSP3 is also associated with protease and ADP-ribose 1”-monophosphatase activities^19^. The functions of other NSPs of TGEV are unknown. The S glycoprotein attaches to the host cellular receptor porcine aminopeptidase N (pAPN) or sialic acid, induces cellular fusion, stimulates neutralizing antibodies, and has hemagglutination activity^20–23^. The pAPN-binding domain of the S protein has two major antigenic sites, A and B^24^. In addition, deletions in the ORF3 gene led to attenuation and reduced pathogenesis of TGEV and *in vivo*^25^.

In 1984, *porcine respiratory coronavirus* (PRCV), which is believed to have evolved from TGEV since PRCV and TGEV shared a high nucleotide percent identity, was first identified in Belgium. Compared to the S gene of TGEV strains, the S gene of PRCV has 621–681 nt deletions at N-terminal, including in strains from Asia^26^ (UF-1 [n = 672 bp]), Europe^7,27^ (AR310 and LEPP [n = 621 bp] and RM4 [n = 672 bp]) and North America^7,25,28^ (OH7269 [n = 648 bp], IA1894 [n = 678 bp], and ISU-1 [n = 681 bp]). Thus, the S gene is used to differentiate PRCV from TGEV. Recently, a novel strain of PRCV (OH7269^28^) was identified in the US, but the origin and evolutionary relationship to current TGEV strains in the US is unknown. While the decline of TGEV is believed to occur in response to partial immunity from PRCV infections^29–32^, the United States swine industry also made significant changes (increased biosecurity, 3 site production model, etc.) to raise healthier pigs, which may have contributed to the reduction of TGEV infections as well.

The porcine enteric coronaviruses (including TGEV, porcine epidemic diarrhea virus [PEDV], and porcine deltacoronavirus [PDCoV]) cause similar clinical presentation, and co-infection of these enteric coronaviruses can occur^33^. In 2010, a highly virulent PEDV emerged in China^34^ and later spread to the US in April 2013^35^. Within less than a year, PDCoV was identified in the US^36,37^. PEDV quickly spread throughout the US^38^, and by March 2014, approximately 41% of the sow herds were infected with PEDV^39^. While the identification of the PEDV lead to an increased biosecurity measure within the US swine industry, prevalence of PEDV in the sow herds did not significantly decrease until July 2014^39^. Within the past couple of years, a chimeric TGEV and PEDV virus (consisting of a TGEV backbone and the spike of PEDV) was identified in multiple countries in Europe^40–42^, illustrating the potential emergence of a chimeric TGEV and PEDV virus in the US.

The occurrence and genetic diversity of TGEV was investigated prior to and after the identification of PEDV and PDCoV in the US from the diagnostic cases submitted to the University of Minnesota Veterinary Diagnostic Laboratory since PEDV recently emerged in the US, and a chimeric TGEV and PEDV virus was identified in Europe. Nineteen TGEV strains (US, n = 18 and Mexico, n = 1) and a single PRCV strain from the US were sequenced, analyzed, and compared with other global TGEV and PRCV strains to characterize historical and currents strains. This research will further our understanding of the occurrence, genetic variability, and evolution of an endemic coronavirus in the US and will provide guidance for future efforts to prevent, monitor, and control endemic coronaviruses.

## Results

### Decline of TGEV positive cases from 2008–2016

**Figure 1:**
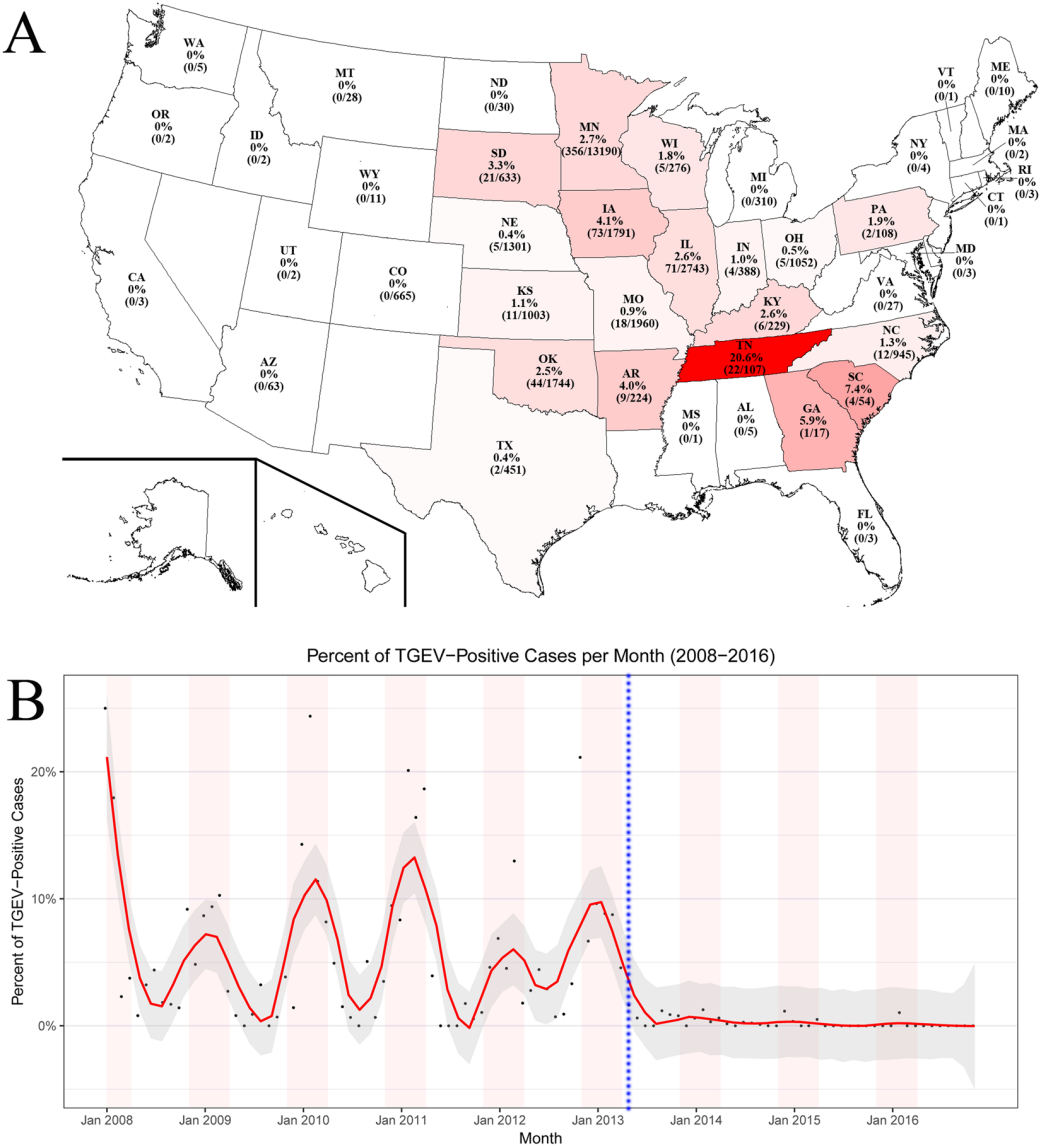
Distribution of TGEV cases. (**A**) The geographical distribution of positive TGEV cases in the US. (**B**) The temporal distribution of positive TGEV cases per month between 2008 to 2016. The black dots represent the percentage of TGEV positive cases per month. A LOESS regression model was fitted to the data (red line), and the shaded area represents the 95% confidence interval. The light red shaded areas represent the cold months of November to April. The blue vertical dotted line represents the introduction of PEDV in April 2013.

Between January 2008 and November 2016, 29,397 porcine enteric cases, distributed across 41 states in the US and Mexico, were tested for TGEV by real time RT-PCR, and 2.3% of the cases (n = 667) were positive for TGEV (Table 1). The percentage of TGEV positive cases was 4.0% in 2008, increased to 6.8% in 2010, and decreased to 0.4% in 2014 (Table 1). After the introduction of PEDV in the US, the prevalence of TGEV decreased further to less than 0.1%. Positive TGEV cases were detected in the main pig raising regions (Midwest, South-central, and Southeast) of the US (Fig. 1A) between January 2008 and November 2016. Most of the cases (n = 13,190) were from Minnesota where 2.7% (n = 356) were positive for TGEV. The percent of positive TGEV cases per state ranged between 0.4–20.6%, with the highest percentage found in Tennessee. A seasonality trend occurred with the positive cases between winter and spring (November to April) compared to summer and fall (May to October) (Fig. 1B).

**Table 1:** Percentage of TGEV positive and negative cases between 2008 to 2016.

Year	Positive Cases		Negative Cases		Total Count
	Count	Percentage	Count	Percentage	
2008	56	4.0%	1,346	96.0%	1,402
2009	64	3.8%	1,605	96.2%	1,669
2010	134	6.8%	1,827	93.2%	1,961
2011	151	6.7%	2,118	93.3%	2,269
2012	119	6.1%	1,839	93.9%	1,958
2013	91	2.1%	4,287	97.9%	4,378
2014	48	0.4%	11,185	99.6%	11,233
2015	2	0.1%	2,297	99.9%	2,299
2016	2	0.1%	2,226	99.9%	2,228
Total	667	2.3%	28,730	97.7%	29,397

### Genomic characteristic, entropy and recombination analyses

**Figure 2:**
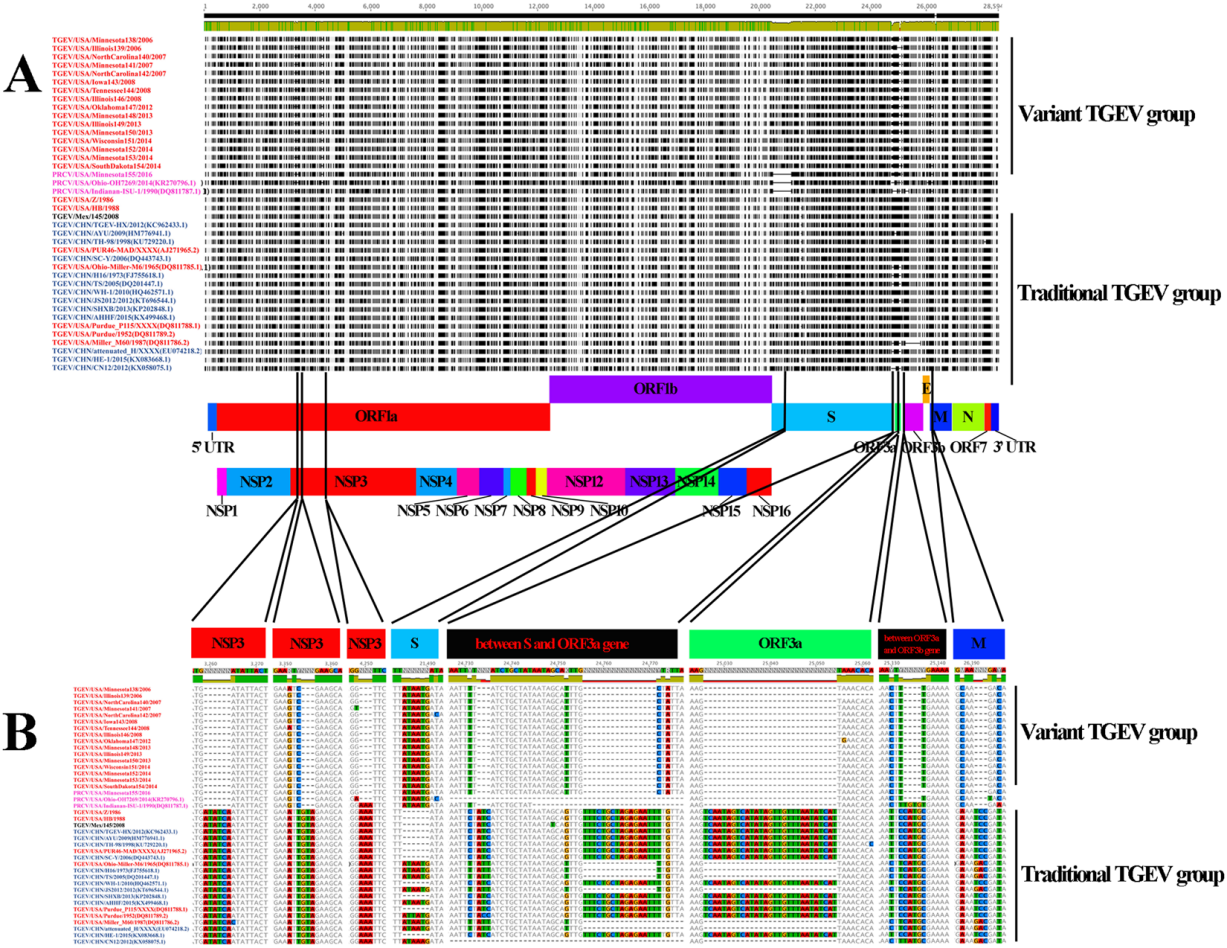
Genomic insertion-deletions (INDELs) within TGEV and PRCV strains. (**A**) Variation within the 30 TGEV and 3 PRCV strains. Thin horizontal dash lines represent deletion in the alignment nucleotides. The vertical black bars represent the differences from consensus sequence. The organization of the genome is listed below (**B**). Visualization of insertion and deletion region of the 30 TGEV and 3 PRCV strains. The dashed lines represent absent nucleotides. The US TGEV strains are colored red. The US PRCV strains are colored purple. The Chinese TGEV strains are colored blue. The Mexican TGEV strain is colored black.

The genomic nucleotide sequence alignments of the 37 TGEV and 3 PCRV strains revealed two main genotypes (traditional and variant genotypes) (Fig. 2A). The traditional genotype shared a genome nucleotide identity of 98.26–100% and contained 21 TGEV strains: 5 US traditional TGEV strains, 2 historical US strains from this study (Z/1986 and HB/1988), the Mexican strain from our study (Mex/145/2008), and 13 Chinese traditional TGEV strains from 1973–2012. Among our 19 TGEV strains, 16 TGEV strains formed a new variant genotype and had a genome nucleotide identity of 98.68–99.97%. The traditional and variant genotypes shared only 95.76–97.13% nucleotide identity. The newly identified PRCV/USA/Minnesota155/2016 had a complete genome nucleotide percent identity of 96.6%, 95.5–96.2%, and 93.1–93.8% with the PRCV, variant TGEV strains, and traditional TGEV strains, respectively.

There were 8 major regions of insertion or deletion (INDELs) between the traditional and variant TGEV strains. In the variant group, 3 deletion regions occurred within NSP3, 2 deletion regions occurred between the S and ORF3a genes, 1 deletion regions occurred in ORF3a genes, 1 deletion region occurred between the ORF3a and ORF3b genes and a single deletion occurred in the M genes (Table 2 and Fig. 2B). Interestingly, the same ORF3a and ORF3b deletions were present in some of the historical TGEV strains. However, these deletions were not present in the TGEV strain Purdue, which was used to create the attenuated TGEV vaccine in the US. The variant TGEV strain Illinois139/2006 had a 128-nucleotide deletion in ORF3a, which resulted in the truncated protein compared with our variant TGEV strains. The traditional TGEV strains Z/1986, HB/1988, and Mex/145/2008 shared the same 6 nucleotide deletions in the S gene that were present in the TGEV strain, Purdue. Surprisingly, the PCRV strains contained an assortment of these deletions in their genomes, and compared to the historical TGEV strains, the TGEV strains from our study (excluding Z/1986, HB/1988 and Mex/145/2008) contained 8 deletions and 119 amino acid changes similar to the recently reported PRCV strain OH7269 and the single PRCV strain Minnesota155/2016 from our study.

**Table 2:** Description of the 8 INDELs between traditional and variant TGEV using TGEV/USA/Z/1986 as reference.

No.	Nucleotide (nt) position	Number of nt deleted	Nt deleted	Correponding amino acid (aa) variation
1	3259–3264	6	ATATCA	Two aa deletion in the NSP3 protein
2	3354–3356	3	GTA	Single aa deletion in the NSP3 protein
3	4249–4251	3	AAA	Single aa deletion in the NSP3 protein
4	24727–24729	3	ATC	Deletion in the non-coding region between S and ORF3a genes
5	24750–24765	16	TTTCTGCTAGAGAATT	Deletion in the non-coding region between S and ORF3a gene
6	25020–25048	29	TCAATAGTCATATAGTTGTTTAATATCAT	Single deletion in the ORF3a protein
7	25127–25130	4	CATG	Deletion in the non-coding region between ORF3a and ORF3b genes
8	26185–26187	3	TCC	Single aa deletion in the M protein

**Figure 3:**
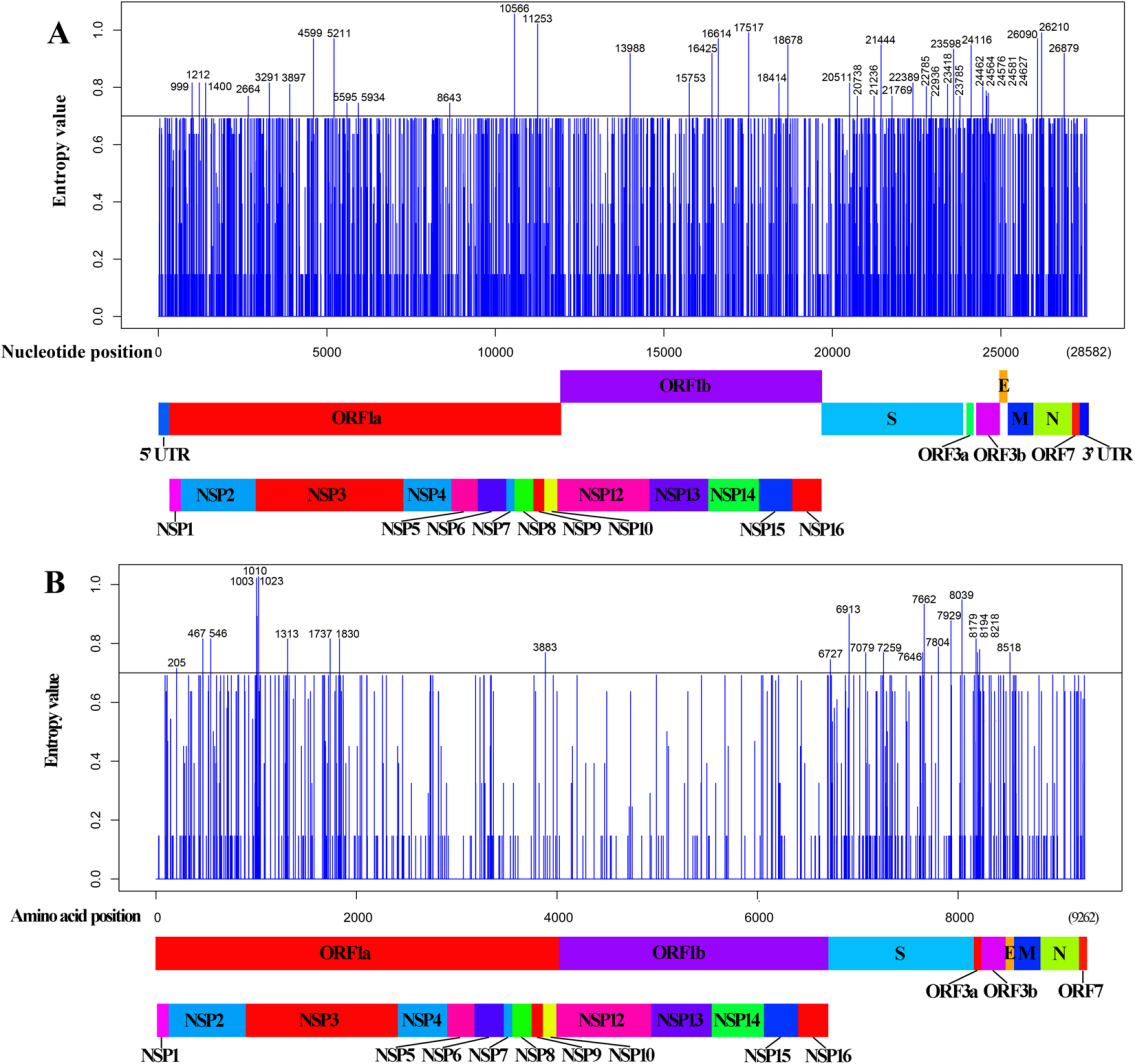
Nucleotide (**A**) and amino acid (**B**) entropy analysis of TGEV strains. The x-axis represents nucleotide or amino acid position while the y-axis represents entropy level. The TGEV non-structural and structural proteins are annotated below each plot. Black line represents the variance threshold of 0.7 for nucleotide and amino acid sequences.

To determine the level of nucleotide or amino acid variation across the TGEV genomes, entropy analysis was conducted with an alignment of the complete genomes and concatenated amino acid sequences (Fig. 3). Based on the level of diversity in the dataset and previous work^43^, entropy values greater than 0.7 were considered highly variable for the nucleotide and amino acid sequence alignments. The ORF1 and S genes had the highest number of nucleotides with entropy levels above 0.7 (n = 20 and n = 12, respectively) while the ORF3b, E and, ORF7 genes lacked diversity in nucleotide positions (Fig. 3A). Within ORF1, the NSP2 and NSP3 had the highest number of nucleotide positions with entropy values greater than 0.7 (n = 4 and n = 6, respectively) while a single position was identified in NSP4, NSP6, NSP8, NSP12, NSP14, and NSP15. Within the amino acid alignment, the M, N and ORF7 genes lacked positions with entropy levels above 0.7 while ORF1 and S proteins (n = 10 and n = 9, respectively) had residues with entropy values higher than 0.7 (Fig. 3). There were 7 high-entropy positions at ORF1b gene in the nucleotide sequence entropy value analysis while entropy value higher than 0.7 were lacking in the amino acid sequence entropy value analysis.

**Figure 4:**
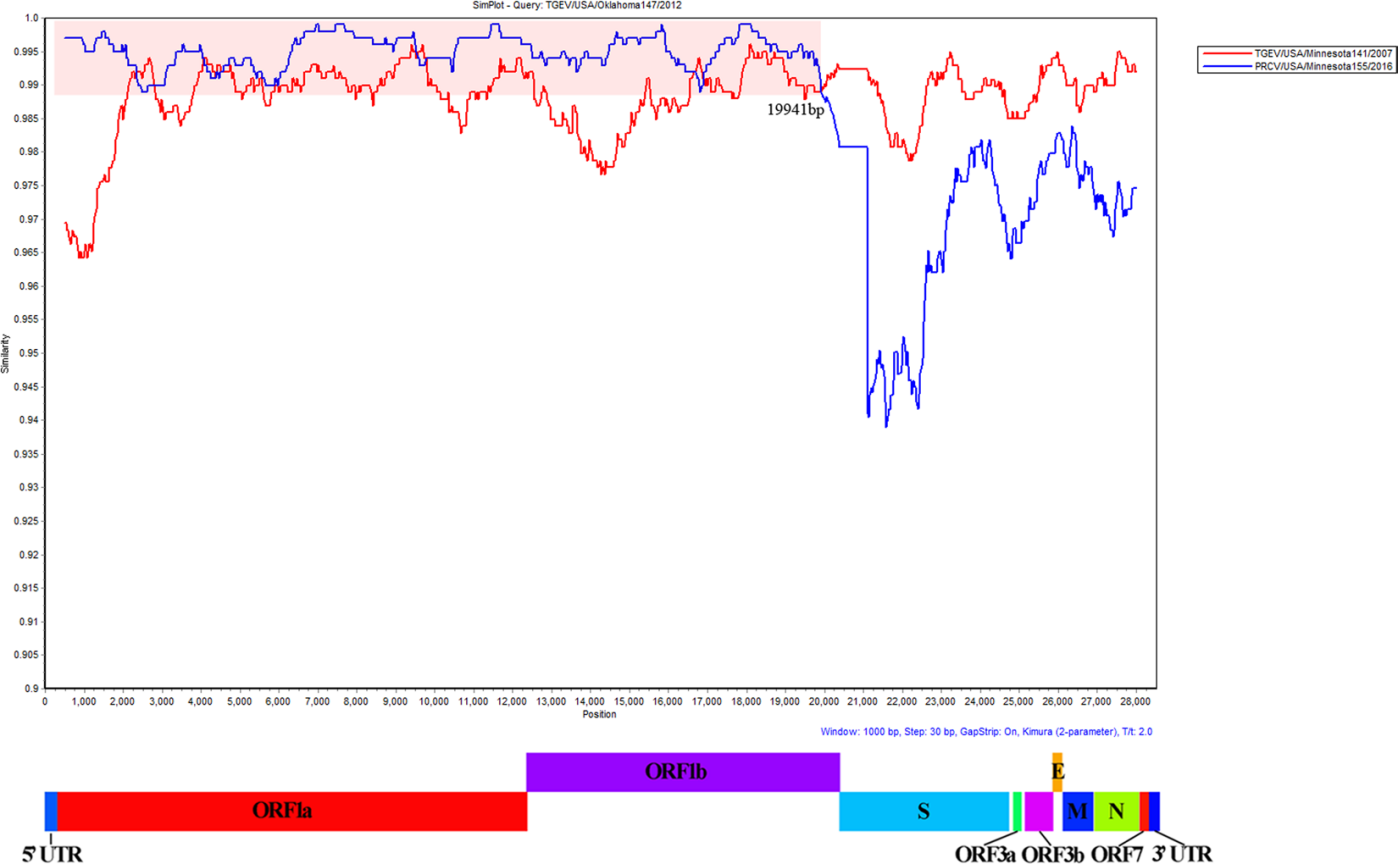
Recombination analysis of the variant TGEV strain Oklahoma147 with Minnesota141/2007 (red colored line) and Minnesota155/2016 (blue colored line). The x and y axes indicate the genomic nucleotide position and genetic identity, respectively. Possible recombination position is shaded with light red, and the breakpoint is marked with nucleotide position number (19,941).

Recombination analysis was performed with the 37 TGEV and 3 PRCV strains. A single recombination event was detected by Chimera, BootScan, MaxChi, and SiScan and RDP within the dataset between the recently identified variant TGEV strains and the novel PRCV strain Minnesota155/2016 (Fig. 4). The TGEV variant Oklahoma147/2012 shares a high nucleotide identity of the first 19,941 nucleotides (breakpoint) with PRCV strain Minnesota155/2016 while the remainder of the genome shares a high nucleotide identity with TGEV strain Minnesota141/2007, indicating a complex nucleotide relationship between PRCV and TGEV.

### Phylogenetic analyses of TGEV and PRCV strains

**Figure 5:**
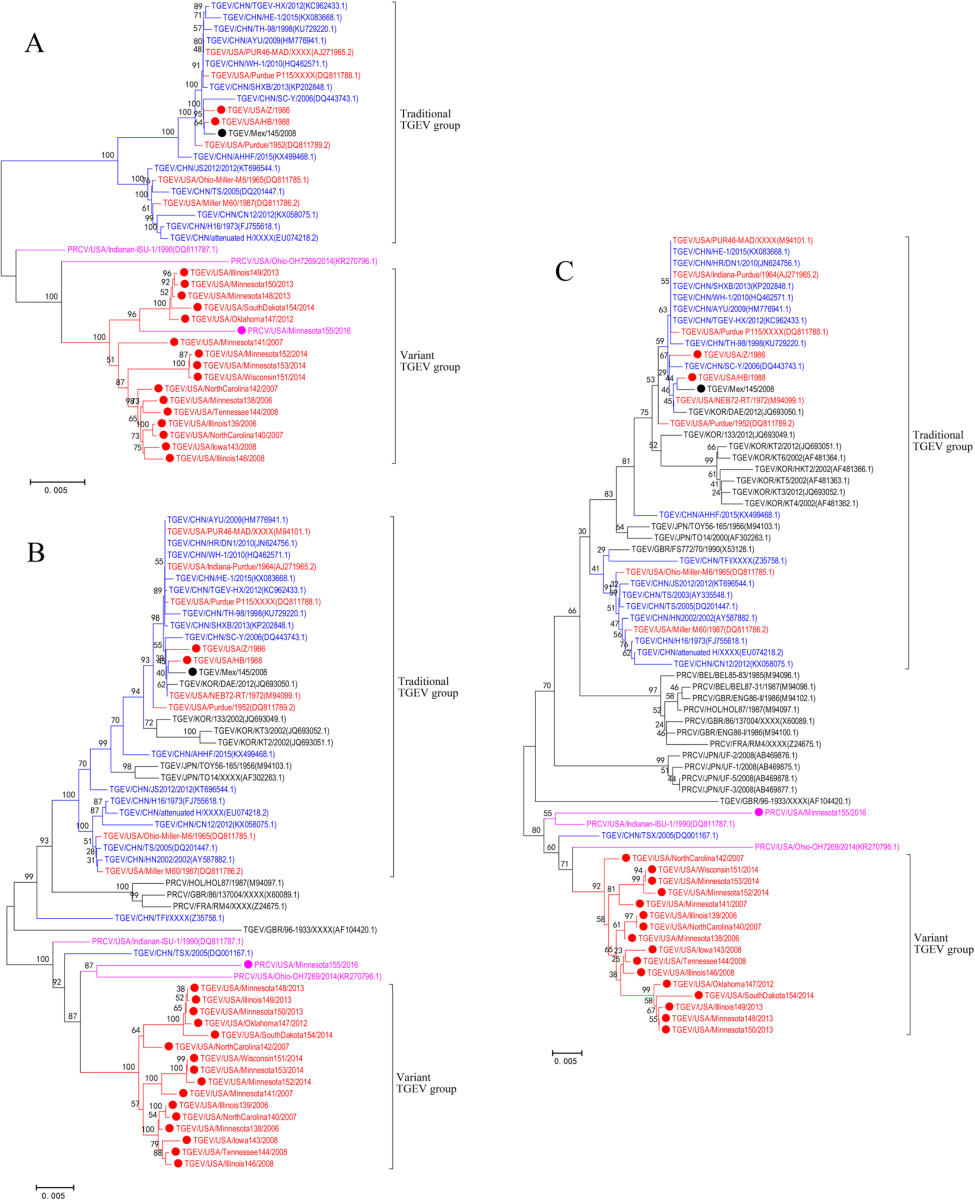
Phylogenetic trees of the TGEV and PRCV strains based on the whole genome (n = 40) (**A**), the whole S gene (n = 56) (**B**), and partial S gene (n = 70) (**C**). The scale bar represents nucleotide substitutions per site. The red color represents the US TGEV strains. The purple color represents the US PRCV strains. The blue color represents Chinese TGEV isolates. The black color represents strains from other countries. Our TGEV or PRCV strains were highlighted by solid circle.

Phylogenetic trees of complete genome sequence (n = 40) and complete S gene (n = 56) revealed two distinct genotypes, representing the traditional and variant TGEV strains from the US (Fig. 5A,B, respectively). In the complete genome and S gene phylogenetic trees, the traditional genotype contained historical strains from the US, the recent strain from Mexico, and historical and recent strains from China. From our study, the 16 recent TGEV strains from the US formed the variant TGEV group, which share a common ancestor with the PRCV strain Ohio-OH7269/2014. Also, the variant TGEV strains were identified in the three main swine production regions of the US (Midwest, South-central, and Southeast) indicating substantial geographical distribution. Interestingly, whole genome phylogenetic analysis of PRCV strain Minnesota155/2016 clustered within the variant genotype and not with historical PRCV strains, indicating significant genetic diversity within PRCV strains circulating in the US.

Since additional partial S gene sequences (first 1383 nt) were available from geographically different locations, a partial S gene phylogenetic tree was constructed to further investigate the global evolutionary relationship between TGEV and PRCV strains (Fig. 5C). The traditional TGEV group consisted of TGEV strains isolated from the US, China, Japan, South Korea, England and Mexico. The variant TGEV group consisted our 16 US TGEV strains isolated in the US after 2006 and a Chinese strain (TSX/2005) from 2005. Interestingly, the Chinese strain TSX/2005 was closely related to the US PRCV strains.

### Analysis of the receptor binding domain

**Figure 6:**
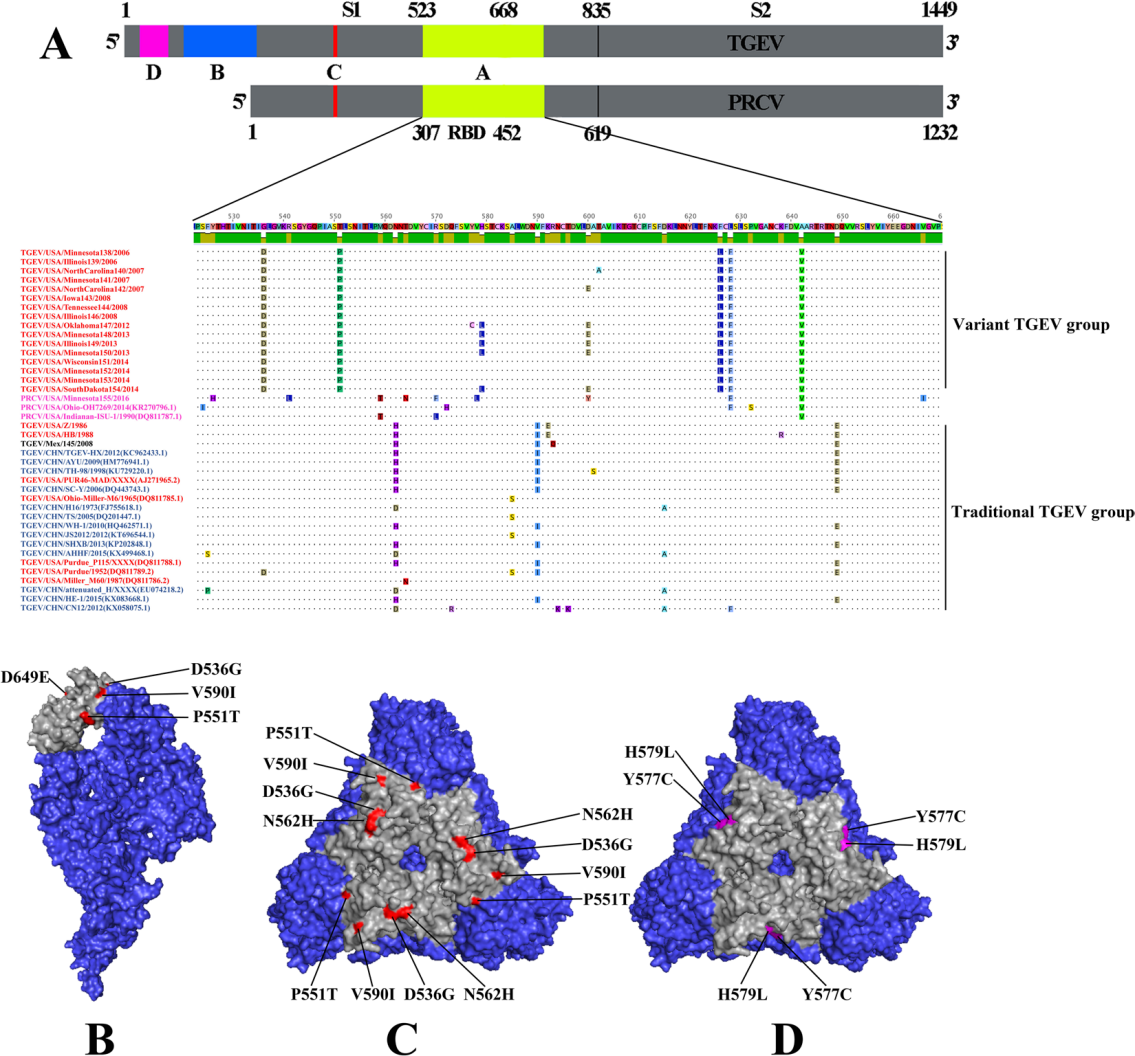
Protein alignment and modeling of S1 domain receptor-binding domain RBD, S protein monomer and trimer. (**A**) The RBD alignment of the 37 whole genome strains and 3 PRCV strains. Identical residues to the consensus sequence are shown as dots while residues that differ from the consensus are highlighted in different colors. (**B**,**C**) The monomer and trimer theoretical protein model of the RBD region, with 4 of the 8 major substitutions highlighted in red. (**D**) Trimer theoretical structure model of RBD, with 2 of the 4 of the TGEV S protein trimer in pink. The body of S protein and the RBD region are highlighted in slate blue and grey.

Given the important role of the S protein in pAPN-receptor binding of TGEV and PRCV, the receptor binding domain (RBD) was investigated for amino acid variation. When comparing the amino acid sequences of the RBD of the traditional to the variant TGEV genotype, eight amino acid substitutions (D536G, P551T, N562H, V590I, L626F, F628L, V642A and D649E) occurred in a majority of the strains while four additional amino acid substitutions (Y577C, H579L, D600E and T602A) were identified in the pAPN (Fig. 6A). The different amino acid between traditional and variant TGEV strains were highlighted in the predicted crystal structure of RBD (Fig. 6B–D). Four of eight amino acid substitutions within the RBD (L626F, F628L, V642A, and D649E) were exposed on the viral protein while two of the four amino acid changes in pAPN (D600E and T602A) were exposed on the viral protein (Fig. 6B–D).

## Discussion

Porcine enteric coronaviruses (PEDV, PDCoV, and TGEV) are significant, emerging pathogens causing severe enteric diseases in the global swine industry. While global historical strains of TGEV caused severe enteritis and PRCV is endemic in Europe, Asia, and the US, the mortality rate due to TGEV infections has declined in these countries^3,44,45^. However, a virulent TGEV or PRCV strain could emerge since the epidemiology of TGEV and PRCV is different in China. In 1992, PRCV was first reported in China^46^ and was not identified or reported in China afterwards while TGEV is constantly reported as a significant viral pathogen for the Chinese pig industry^10,15,16^. The different clinical status of TGEV in China and the western countries could be due to the stringent biosecurity measures in western countries compared to China. TGEV was consistently detected in the US between 2008 and 2016, but after the introduction of PEDV into the US, the swine industry significantly increased their biosecurity to prevent and control PEDV infections and the prevalence of PEDV were reduced after March 2014 in US^39,47,48^, which indirectly may have reduced the prevalence of TGEV to less than 0.1%. Our study illustrates a seasonality pattern with TGEV in the US with infection peaking during cold months, which has been identified in our swine pathogens as well^37,45,46^.

While TGEV has been identified throughout Europe^1,8,49,50^, Asia^11,15,16,45^, and North America^5,6^, only the variant TGEV genotype was detected in US pig farms since 2006, suggesting that variant TGEV is the dominant genotype currently circulating in the main pig raising Midwest, South-central, and Southeast regions. In addition, the first TGEV genome from Mexico was characterized, which helps us to understand the evolutionary relationship of TGEV strains from different countries. Surprisingly, the Mex/148 strain is phylogenetically related to the traditional TGEV, and not the variant TGEV strain. Given the close geographical proximity of Mexico and US and that the Mexican and US PEDV strains are phylogenetically related^51^, the variant TGEV strains could be circulating in Mexico, but were not represented in our study since our study had only a single TGEV strain from Mexico. The circulation of TGEV and PEDV in the US indicates a chimeric virus could emerge in the US similar as the chimeric virus emerged in Europe. Characterizing of the current TGEV strains will aid in understanding the emergence of virulent or chimeric TGEV strains in the US, if such an event would occur in the future.

PRCV was first identified in Belgium in 1984^44^ and has been identified multiple countries including Belgium^52^, China^53^, Japan^26^, Uganda^12^ and the US^37^. The evolutionary relationship between the recently identified PRCV strain (Ohio-OH7269/2014) and our PRCV strain (Minnesota155/2016) was unclear given the limited number of globally available PRCV strains. Ideally, additional PRCV strains would have been sequenced, but routine screen of PRCV does not occur since PRCV is not considered as a significant pathogen in swine. The lab accidently isolated the PRCV strain, which was added to the study since the genetic and evolutionary relationship of PRCV is unknown in the US. The variant TGEV strains shared the same nucleotide mutations and amino acid deletions with the novel PRCV strain Ohio-OH7269/2014, and a recombination event between PRCV and TGEV was identified, illustrating the complex evolutionary relationship between TGEV and PRCV strains in the US. Hopefully, future US studies will assess the genetic and evolutionary of PRCV strains to fully elicit the complex relationship with TGEV.

Historically, a partial gene fragment or a single gene of TGEV was sequenced^6^ to differentiate viral strains due to limitations in technology and economic constraints. In this study, NGS technology generated the whole TGEV genome, revealing a total of 8 unique INDELs located in NSP3, S, ORF3a, ORF3b and M protein, and these regions, excluding the M protein, had high entropy levels in our study (Fig. 3A). The NSP3, S, ORF3a and ORF3b proteins of TGEV are associated with enteric tropism, immunogenicity, neutralization, sialic acid and other receptor binding activity ability, virulence, protease and ADP-ribose 1 ″-monophosphatase activities^18–25^, and changes in these proteins might reduce INDELs efficacy to cause clinical disease. Mutations in the spike gene in other coronaviruses has impact on initiation of membrane fusion^54^, sialic acid binding activity^22^, confer resistance to virus neutralization^55^, and render trypsin independent for cell entry and fusion^56^, which could be contribution to a reduction of the overall fitness of the INDELs. The attenuation of murine coronavirus in the natural host occurred due to deletion of the HE, 2a/HE, and 4/5a genes^57^, epidemics of feline infectious peritonitis were contributed to deletions in the ORF7a and ORF7b of feline coronavirus^58,59^, and disruption of functional expression of accessory protein occurred due to deletion of ORF8 in human severe acute respiratory syndrome coronaviruses^60^. Given the results from the previous studies, we hypothesis the INDELs are significantly attenuated compared to the traditional strains of TGEV. Further studies are needed to confirm this hypothesis and investigate the effect of these deletions and mutations on the biological characteristics and fitness of the new TGEV genotype.

In conclusion, a variant genotype of TGEV is the predominant in the US and evolutionary relationship between TGEV and PRCV is complex. The decline of TGEV positive diarrhea cases after May 2013 in the US may be indirectly associated with the outbreak of PED in the US via increased biosecurity. Compared with the traditional TGEV strains, the unique amino acid INDELs might affect the biological characteristics of the variant TGEV, which could lead to changes in pathogenesis or chimeric virus in the future.

## Materials and Methods

Veterinarians routinely send samples to the University of Minnesota Veterinary Diagnostic Laboratory (MNVDL) to determine potential pathogenic agents contributing to disease and to promote the health of swine herds. The samples may represent clinical outbreaks of diarrhea or are for routine monitoring of enteric pathogens in swine herds. Upon arrival, ownership of the samples belongs to the MNVDL and client(s) confidentiality is retained by removing identifiers associated with client(s) information. Between January 2008 and November 2016, a total of 29,397 samples, including fresh intestines and fecal samples from of clinical cases were submitted from 41 US states and Mexico and tested for TGEV by real time RT-PCR under Standard Operation Procedures (available upon request) and various bacterial and viral enteric pathogens dependent on the veterinarian’s request. Request may include beta-hemolytic *Escherichia coli*, non-beta-hemolytic *Escherichia coli*, *rotavirus A, B, and C*, PEDV, and PDCoV. Randomly selected historical positive TGEV samples from the MNVDL (n = 17) were saved from previous enteric studies, two TGEV samples from Ohio (Z and HB) were supplied from Dr. Linda Saif. Routine testing for PRCV does not occur since it is not considered a major swine pathogen. However, a PRCV isolate from MN was obtained from a nasal swab in 2016 during an attempted to isolate other viruses. All samples (n = 20) were selected for whole genome sequencing using next generation sequencing as previously described^36^. TGEV prevalence information was exported from the MNVDL database and analyzed at the case-level with R software^61^ using the ggplot2^62^ and maps^63^.

To investigate the differences in TGEV strains and phylogenetic relationship with PRCV, our 20 sequences and available sequences from GenBank (Table S1) were aligned using ClustalW in Geneious v9.1.4^64^. Nucleotide and amino acid entropy analyses of the concatenation ORFs (ORF1a/b, S, ORF3a/3b, envelope, membrane, nucleocapsid and ORF7) was performed using the MATLAB^65^ to determine regions of diversity within the alignment. Entropy values higher than 0.7 in the nucleotide and amino acid alignments were identified as high variation positions^43^. Recombination analysis was performed using Recombination Detection Program (RDP)^66^ v4 with RDP, BootScan, GEENECOV, SiScan and MAXCHI algorithms (Window size is 100 bp). Recombination event was represented using similarity plot, with Oklahoma147 as the query strain. The similarity plot was implemented in the SimPlot, v. 3.5.1 package^67^, using the two-parameter (Kimura) distance model with a sliding window of 1000 bp and step size of 30 bp.

Whole genome (n = 40), the whole S gene (n = 56), and the partial S gene (first 1383 nt) (n = 70) (Table S1) phylogenetic trees were constructed using the Maximum likelihood algorithm, with a GTR nucleotide substitution model (bootstrap analysis with 1,000 replicates) by MEGA v6.06^68^. The S protein receptor binding domains (RBDs) of TGEV and PRCV were modeled using the open-source modeling server SWISS-MODEL^69^ provided by the Swiss Institute of Bioinformatics. Predicted tertiary structure of the RBDs of TGEV and PRCV were modeled using PRCV RBD (PDB accession no. 5SZS) reported in the previous study. Spike monomer and trimer models were performed using human coronavirus NL63 model as a template to theatrically visualize the changes in the residues since TGEV and PRCV templates are lacking^70^. Illustrations were created using the Python-based molecular viewer PyMOL^71^.

## Supplementary information

Supplemental Table 1
