# Supplementary material for: Decline of transmissible gastroenteritis virus and its complex evolutionary relationship with porcine respiratory coronavirus in the United States
Source: Sci Rep. 2019 Mar 8;9:3953. doi: 10.1038/s41598-019-40564-z (PMC6408454; doi:10.1038/s41598-019-40564-z)
Supplement: Supplementary file 1 — Supplemental Table 1 [file 41598_2019_40564_MOESM1_ESM.docx]

| **Table S1. The TGEV and PRCV strains used in this study.** | |  |  |  |
| --- | --- | --- | --- | --- |
| No. | Strain Name | GenBank accession no. | Location | Collection date |
| 1 | TGEV/USA/Z/1986 | KX900393 | United States | 1986 |
| 2 | TGEV/USA/HB/1988 | KX900394 | United States | 1988 |
| 3 | TGEV/USA/Minnesota138/2006 | KX900395 | United States: Minnesota | 11/8/06 |
| 4 | TGEV/USA/Illinois139/2006 | KX900396 | United States: Illinois | 11/17/06 |
| 5 | TGEV/USA/North Carolina140/2007 | KX900397 | United States: North Carolina | 2/8/07 |
| 6 | TGEV/USA/Minnesota141/2007 | KX900398 | United States: Minnesota | 2/8/07 |
| 7 | TGEV/USA/North Carolina142/2007 | KX900399 | United States: North Carolina | 2/16/07 |
| 8 | TGEV/USA/Iowa143/2008 | KX900400 | United States: Iowa | 3/6/08 |
| 9 | TGEV/USA/Tennessee144/2008 | KX900401 | United States: Tennessee | 4/15/08 |
| 10 | TGEV/Mex/145/2008 | KX900402 | Mexico | 4/17/08 |
| 11 | TGEV/USA/Illinois146/2008 | KX900403 | United States: Illinois | 4/9/08 |
| 12 | TGEV/USA/Oklahoma147/2012 | KX900404 | United States: Oklahoma | 11/29/12 |
| 13 | TGEV/USA/Minnesota148/2013 | KX900405 | United States: Minnesota | 1/4/13 |
| 14 | TGEV/USA/Illinois149/2013 | KX900406 | United States: Illinois | 2/28/13 |
| 15 | TGEV/USA/Minnesota150/2013 | KX900407 | United States: Minnesota | 3/1/13 |
| 16 | TGEV/USA/Wisconsin151/2014 | KX900408 | United States: Wisconsin | 1/17/14 |
| 17 | TGEV/USA/Minnesota152/2014 | KX900409 | United States: Minnesota | 1/31/14 |
| 18 | TGEV/USA/Minnesota153/2014 | KX900410 | United States: Minnesota | 2/4/14 |
| 19 | TGEV/USA/South Dakota154/2014 | KX900411 | United States: South Dakota | 2/5/14 |
| 20 | PRCV/USA/Minnesota155/2016 | KY406735 | United States: Minnesota | 11/6/16 |
| 21 | PRCV/USA/Ohio-OH7269/2014 | KR270796 | United States | 2014 |
| 22 | PRCV/USA/Indianan-ISU-1/1990 | DQ811787 | United States | 1990 |
| 23 | TGEV/USA/Indiana-Purdue/1964 | AJ271965 | United States | 1964 |
| 24 | TGEV/USA/Ohio-Miller-M6/1965 | DQ811785 | United States | 1965 |
| 25 | TGEV/CHN/TGEV-HX/2012 | KC962433 | China | 2012 |
| 26 | TGEV/CHN/AYU/2009 | HM776941 | China | 2009 |
| 27 | TGEV/CHN/TH-98/1998 | KU729220 | China | 1998 |
| 28 | TGEV/CHN/SC-Y/2006 | DQ443743 | China | 2006 |
| 29 | TGEV/CHN/H16/1973 | FJ755618 | China | 1973 |
| 30 | TGEV/CHN/WH-1/2010 | HQ462571 | China | 2010 |
| 31 | TGEV/CHN/JS2012/2012 | KT696544 | China | 2012 |
| 32 | TGEV/CHN/SHXB/2013 | KP202848 | China | 2013 |
| 33 | TGEV/USA/NEB72-RT/1972 | M94099 | United States | 1972 |
| 34 | TGEV/JPN/TOY56-165/1956 | M94103 | Japan | 1956 |
| 35 | TGEV/KOR/KT3/2012 | JQ693052 | South Korea | 2012 |
| 36 | TGEV/KOR/KT2/2012 | JQ693051 | South Korea | 2012 |
| 37 | TGEV/KOR/DAE/2012 | JQ693050 | South Korea | 2012 |
| 38 | TGEV/KOR/133/2012 | JQ693049 | South Korea | 2012 |
| 39 | TGEV/CHN/HN2002/2002 | AY587882 | China | 2002 |
| 40 | TGEV/CHN/TS/2003 | AY335548 | China | 2003 |
| 41 | TGEV/CHN/TSX/2005 | DQ001167 | China | 2005 |
| 42 | TGEV/CHN/HR/DN1/2010 | JN624756 | China | 2010 |
| 43 | TGEV/JPN/TO14/2000 | AF302263 | Japan | 2000 |
| 44 | TGEV/GBR/FS772/70/1990 | X53128 | United Kingdom | 1990 |
| 45 | PRCV/HOL/HOL87/1987 | M94097 | Netherlands | 1987 |
| 46 | TGEV/KOR/HKT2/2002 | AF481366 | South Korea | 2002 |
| 47 | TGEV/KOR/133/2002 | AF481365 | South Korea | 2002 |
| 48 | TGEV/KOR/KT6/2002 | AF481364 | South Korea | 2002 |
| 49 | TGEV/KOR/KT5/2002 | AF481363 | South Korea | 2002 |
| 50 | TGEV/KOR/KT4/2002 | AF481362 | South Korea | 2002 |
| 51 | PRCV/BEL/BEL87-31/1987 | M94098 | Belgium | 1987 |
| 52 | PRCV/BEL/BEL85-83/1985 | M94096 | Belgium | 1985 |
| 53 | PRCV/GBR/ENG86-II/1986 | M94102 | United Kingdom | 1986 |
| 54 | PRCV/GBR/ENG86-I/1986 | M94100 | United Kingdom | 1986 |
| 55 | PRCV/JPN/UF-5/2008 | AB469878 | Japan | 2008 |
| 56 | PRCV/JPN/UF-3/2008 | AB469877 | Japan | 2008 |
| 57 | PRCV/JPN/UF-2/2008 | AB469876 | Japan | 2008 |
| 58 | PRCV/JPN/UF-1/2008 | AB469875 | Japan | 2008 |
| 59 | PRCV/GBR/86/137004/XXXX | X60089 | United Kingdom | N/A |
| 60 | PRCV/FRA/RM4/XXXX | Z24675 | France | N/A |
| 61 | TGEV/USA/PUR46-MAD/XXXX | M94101 | United States | N/A |
| 62 | TGEV/GBR/96-1933/XXXX | AF104420 | United Kingdom | N/A |
| 63 | TGEV/CHN/TFI/XXXX | Z35758 | China | N/A |
| 64 | TGEV/CHN/AHHF/2015 | KX499468 | China | 2015 |
| 65 | TGEV/USA/Purdue_P115/XXXX | DQ811788 | United States | N/A |
| 66 | TGEV/USA/Purdue/1952 | DQ811789 | United States | 1952 |
| 67 | TGEV/USA/Miller_M60/1987 | DQ811786 | United States | 1987 |
| 68 | TGEV/CHN/attenuated_H/XXXX | EU074218 | China | N/A |
| 69 | TGEV/CHN/HE-1/2015 | KX083668 | China | 2015 |
| 70 | TGEV/CHN/CN12/2012 | KX058075 | China | 2012 |

**Decline of transmissible gastroenteritis virus and its complex evolutionary relationship with porcine respiratory coronavirus in the United States**

Fangzhou Chen, Todd P. Knutson, Stephanie Rossow, Linda J. Saif & Douglas G. Marthaler
